# Protective effect of melatonin against methotrexate-induced testicular damage in the rat model: An experimental study

**DOI:** 10.18502/ijrm.v13i5.7153

**Published:** 2020-05-31

**Authors:** Wannisa Sukhorum, Jariya Umka Welbat, Suchada Krutsri, Sitthichai Iamsaard

**Affiliations:** ^1^School of Medicine, Mae Fah Luang University, Chiang Rai, Thailand.; ^2^Department of Anatomy, Faculty of Medicine, Khon Kaen University, Khon Kaen, Thailand.; ^3^Research Institute for Human High Performance and Health Promotion (HHP & HP), Khon Kaen, Thailand.

**Keywords:** Melatonin, Testis, Sperm, Methotrexate, Caspase-3, Tyrosine phosphorylation.

## Abstract

**Background:**

Methotrexate (MTX) has been shown to affect the testes adversely, especially the seminiferous epithelium. As melatonin, an endocrine hormone, has been shown to normalize testicular function, its ability to prevent MTX-induced testicular damage should be considered.

**Objective:**

Based on the antioxidant, anti-inflammatory, and antiapoptotic activities of melatonin, this study aimed to investigate its protective effect against testicular damage induced by MTX.

**Materials and Methods:**

Forty adult male rats (200-230 g) were divided into five groups (n = 8/each). The rats in group I were injected with vehicle as a control. In group II, the rats were received intraperitoneal injections of melatonin (8 mg/kg) for 15 consecutive days. The rats in group III were intravenously injected with MTX (75 mg/kg) for 15 consecutive days. The remaining two groups received melatonin (8 mg/kgBW) for 15 (group IV) and 30 (group V) consecutive days, intraperitoneally, and then intravenously received MTX (75 mg/kgBW) on days 8 and 15 of the experimental period. Reproductive parameters, including epididymal sperm concentration, testicular tyrosine-phosphorylated protein expression, steroidogenic acute regulatory (StAR) protein expression, and caspase-3 and malondialdehyde levels, were examined.

**Results:**

The sperm concentrations (×10${}^{6}$/ml) of groups IV (58.75 ± 1.28) and V (55.93 ± 2.57) were improved significantly (p = 0.032) compared with that of group II (32.92 ± 2.14). The seminiferous epithelium in groups IV and V also increased, while caspase-3 expression decreased. In the melatonin-treated groups, the expression of tyrosine-phosphorylated proteins at 32 kDa was decreased and that of proteins at 47 kDa was increased compared with the MTX group. StAR protein expression was not altered in any of the groups.

**Conclusion:**

Our results indicate that melatonin improves the epididymal sperm concentration by decreasing the expression of caspase-3 and increasing that of tyrosine-phosphorylated proteins in MTX-treated testes.

## 1. Introduction

Many reports have demonstrated that drugs used for chemotherapy have side effects on several organs, including those of the reproductive system (1, 2). In the male reproductive system, some chemotherapeutic agents have been reported to cause azoospermia, testicular damage, sex hormone dysfunction, and infertility in human and animal models due to their anti-cell division and antiproliferative activity (3, 4). Methotrexate (MTX), a folic acid antagonist, is used widely to treat various neoplastic and non-malignant diseases, such as rheumatoid arthritis, psoriasis, graft-versus-host disease, multiple sclerosis, and keratoacanthomas (5, 6). However, MTX has been reported to have negative side effects on the male reproductive system (7-9). Previous studies have indicated that MTX causes disorganization and vacuolization of the seminiferous epithelium, decreased sperm count, damaged sperm DNA, and decreased testicular weight as well as the seminal vesicle and prostate gland (10-12). Moreover, MTX causes an increase in the number of apoptotic cells and the oxidative stress in the testes (9, 13). Recently, MTX was shown to affect the expression of testicular functional proteins, such as tyrosine-phosphorylated and steroidogenic acute regulatory (StAR) proteins (14). Moreover, MTX may induce testicular germ cell apoptosis by increasing the apoptotic index and the mRNA expression of caspase-3, caspase-8, and caspase-9 (8, 15). Further, the adverse effects of MTX have been associated with an increase in reactive oxygen species (ROS) in the testes (15). Numerous reports have also found increased levels of malondialdehyde (MDA) and low superoxide dismutase (SOD) and catalase (CAT) activity in the testicular tissue of MTX-treated animals (15, 16).

Melatonin is an endocrine hormone produced by the pineal gland to regulate normal testicular function through the hypothalamic-adenohypophyseal axis (17, 18). Additionally, melatonin has been shown to have anti-inflammatory and proliferative effects (18, 19). Moreover, it has been demonstrated to reduce the expression of caspase-3 in ovaries and testes damaged by chemotherapeutic drugs (20-23). Based on the antioxidant, anti-inflammatory, and antiapoptotic activities of melatonin, the present study aimed to investigate its protective effect against testicular damage induced by MTX.

## 2. Materials and Methods

### Animals and experiment design

The male reproductive organs of rats were obtained from a research project led by associate professor, Dr. Jariya Umka Welbat (Department of Anatomy, Faculty of Medicine, Khon Kaen University). The research was approved by the Animal Ethics Committee of Khon Kaen University, based on the Ethics of Animal Experimentation of Thailand (Record No. ACU-KKU-46/2559). Briefly, the 40 adult male Sprague-Dawley rats (200-230 g) were purchased from the National Laboratory Animal Center (Salaya, Nakhon Pathom, Thailand). The animals were housed under standard conditions in the North-East Laboratory Animal Center at Khon Kaen University in Thailand. The rats were divided into five groups (n = 8/each):

• Group I (control group) received an ethanol and normal saline solution similar to the treated groups.

• Group II (melatonin-treated group) received intraperitoneal injections of melatonin (Sigma-Aldrich, Inc., St. Louis, MO, USA) at a dose of 8 mg/kg for 15 consecutive days.

• Group III (MTX group) were intravenously injected with 0.5 ml/kg MTX (Pharmachemie B.V., Harsblem, the Netherlands) at a dose of 75 mg/kg on days 8 and 15 of the experiment. They were also intraperitoneally injected with leucovorin (Ben Venue Laboratories, Inc., Bedford, MA, USA) at a volume of 1 ml/kg (at a dose of 6 mg/kg) 18, 26, 42, and 50 hour after the MTX injections,

• Group IV were intraperitoneally injected with melatonin (8 mg/kg) for 15 consecutive days and MTX (75 mg/kg) on days 8 and 15 of the experimental period.

• Group V were intraperitoneally injected with melatonin (8 mg/kg) for 30 consecutive days and MTX (75 mg/kg) on days 8 and 15 of the experimental period.

Melatonin was freshly prepared by dissolution in ethanol and dilution with 0.9% normal saline solution. At the end of the experiment, animals were euthanized by rapid stunning and cervical dislocation as described previously (24).

### Histological examination of male reproductive organs and sperm concentration

At the end of the experiment, the testes, epididymis plus vas deferens, and seminal vesicle plus prostate gland were collected, and the fat pads surrounding the organs were removed gently. The left testis and epididymis plus vas deferens were then fixed rapidly in 10% formalin and processed for paraffinization before being sectioned at 5-μm thickness. All tissue sections were stained with hematoxylin and eosin dyes. The histology of the testis and epididymis was observed and captured by a Nikon ECLIPSE E200 microscope equipped with a DXM1200 digital camera. For seminiferous tubule (ST) diameter measurement, the diameters of 10 randomly selected ST cross-sections from each animal were measured. Each selected ST was measured for the distance from the basement membrane to the opposite basement membrane in four planes using the ImageJ 1.52a program (4). The sperm mass and fluid from the right cauda epididymis and vas deferens were squeezed and dipped into 1 ml phosphate-buffered saline (PBS; pH 7.4). Subsequently, the sperm suspension was diluted (1:10), loaded onto a hemocytometer, and counted under a microscope. The sperm concentration (×10${}^{6}$/ml) was calculated as explained previously (25).

### Activated caspase-3 immunofluorescence 

The paraffinized testicular blocks were sectioned to 5 μm using a rotary microtome (Leica RM2235) in a preclinic building at the School of Medicine of Mae Fah Luang University. The testicular sections were placed on gelatin-coated glass slides and warmed in a hot air oven (55-60°C). The sections were then deparaffinized with xylene and rehydrated with serial alcohols. In the antigen retrieval step, the sections were soaked twice and immersed in citrate buffer (10 mM citric acid, 0.05% Tween-20, pH 6.0) in a microwave at a high temperature (800 W) for 5 min. After washing the citrate buffer, the sections were then blocked for non-specific proteins by incubation with 5% bovine serum albumin (BSA) in PBS (pH 7.4) for 20 min in a moist chamber in darkness. To detect caspase-3 protein, all sections were incubated with monoclonal anti-caspase-3 antibody (Santa Cruz Biotechnology) diluted with PBS (1:50) in a moist chamber at 4°C overnight. Then, the non-specific binding antibodies in all sections were washed out with PBS and incubated with anti-mouse IgGκ light chain-binding protein (m-IgGκ BP) conjugated with fluorescein isothiocyanate (FITC; 1:50; Santa Cruz Biotechnology) in a moist chamber for 1 h at room temperature. Finally, the caspase-3 protein-antibody complexes were detected on each section by FITC luminescence emission under a Carl Zeiss fluorescence microscope (AxioScope; Rushmore Precision Co., Ltd., city, state code, country) via the ZEN 2.3 (blue edition) program. The caspase-3 complex gave a green fluorescence positive signal.

### Western blot analysis

The testicular protein samples were extracted with radioimmunoprecipitation (RIPA) buffer (Cell Signaling Technology, Inc., city, state code, USA) containing a protease inhibitor cocktail (Sigma-Aldrich, Inc.). The supernatant testicular lysate was then measured for total protein concentration using a NanoDrop ND-1000 Spectrophotometer (Nano Drop Technology, Inc., city, state code, USA) at an absorbance of 280 nm in the Vejawichakarn building of the Faculty of Medicine at Khon Kaen University. The testicular protein profile (80 µg) was separated on 12% separating gel (SDS-PAGE).

The separated proteins were transferred onto a nitrocellulose membrane (Bio-Red Laboratories, Inc., city, Germany) in a 10% methanol transfer buffer. Subsequently, the non-specific proteins were blocked with 5% skim milk. To examine the expression of tyrosine-phosphorylated protein, StAR protein, and caspase-3, the membrane was incubated with monoclonal anti-phosphotyrosine 4G10 antibody (1:1000; Millipore, city, state code, country), polyclonal anti-StAR antibody (1:2000; Santa Cruz Biotechnology), or monoclonal anti-caspase-3 antibody (1:100; Santa Cruz Biotechnology) at 4°C overnight. The membrane was also incubated with monoclonal anti-β-actin antibody as an internal control (1:1000; Santa Cruz Biotechnology).

Each of the non-binding antibodies was washed out of the membrane, and the membrane was further incubated with a non-binding antigen-antibody complex for each antibody. The complexes were composed of specific secondary antibodies conjugated to the primary antibodies with horseradish peroxidase at room temperature. After washing, each non-binding antigen-antibody complex was incubated with an enhanced chemiluminescence substrate kit reagent (GH Healthcare Life Science). The positive protein bands were detected using the ImageQuant LAS 500 imaging system (GH Healthcare Life Science) in the Vejawichakarn building of the Faculty of Medicine at Khon Kaen University in Thailand.

To quantify the level of target protein expression, the intensities of protein bands were measured using the Image J 1.52a program. In this study, BSA (Millipore), epidermal growth factor (Millipore), and StAR lysate (Santa Cruz Biotechnology) were used as specific negative and positive controls of tyrosine-phosphorylated protein and StAR protein, respectively (4).

### MDA examination

The testicular protein was extracted using RIPA buffer (Cell Signaling Technology, Inc.) containing a protease inhibitor cocktail (Sigma-Aldrich, Inc.). The testicular tissue was then homogenized using a glass grinder and an ultrasonic homogenizer (Cole-Parmer, 04714-53, Vernon Hills, IL, USA; microprocessor-controlled 100-W model). To separate the testicular supernatant from cell debris, the tissue homogenate was ultracentrifuged at 14,000 rpm. The supernatants were then measured for total protein concentration using the NanoDrop spectrophotometer. Subsequently, the MDA level in the supernatant was measured using the thiobarbituric acid reactive substance assay as explained previously (4). Briefly, the principle of the assay is to detect the reaction between testicular MDA molecules and thiobarbituric acid, the product of which turns into a pink chromogenic compound that can be measured at an absorbance of 540 nm (Sunrise absorbance reader, Tecan, Männedorf, Switzerland). The testicular MDA levels were expressed as nmol of MDA/mg of testicular protein.

### Ethical consideration

This research was approved by Animal Ethics Committee of Khon Kaen University, based on the Ethics of Animal Experimentation of National Research Council of Thailand (Record No. ACU-KKU-46/2559, Ref. No. 0514.1.75/51)

### Statistical analysis

The data analysis was carried out using the Statistical Package for the Social Sciences (SPSS software, Chicago, Illinois), version 22.0. To determine the significant differences between groups, One-way analysis of variance and Student's t tests were used. P-value < 0.05 was considered statistically significant.

## 3. Results

### Effects of melatonin on the body weight and reproductive parameters of male rats treated with MTX

The results revealed no significant difference in body weight among groups (Table I). However, the weights of the testes and epididymis plus vas deferens of the melatonin group were higher than those of the MTX group. Compared with the control and melatonin groups, the sperm concentration in the MTX group was also low (Table I). The preventive (IV and V) and melatonin groups both showed improvements in the epididymal sperm concentration compared with the MTX group (Table I). Moreover, in comparison with the MTX group, the ST diameter of the melatonin group was improved significantly (Table I).

### Effects of melatonin on the histology of the testes and cauda epididymis in the MTX group

The results revealed that the melatonin did not *had* a significant effect on the histology of the testes and cauda epididymis in the MTX group compared with the control. However, reductions in the seminiferous epithelium (Table I) and epididymal sperm mass were observed in the MTX group. Additionally, the MTX group showed wide interstitial spaces compared with the control group. Both the preventive (IV and V) and melatonin groups showed normal seminiferous epithelium compared with the control and melatonin groups. No differences in epididymal histology were found among any of the groups.

### Effects of melatonin and MTX on testicular tyrosine-phosphorylated protein

**Figure 1:**
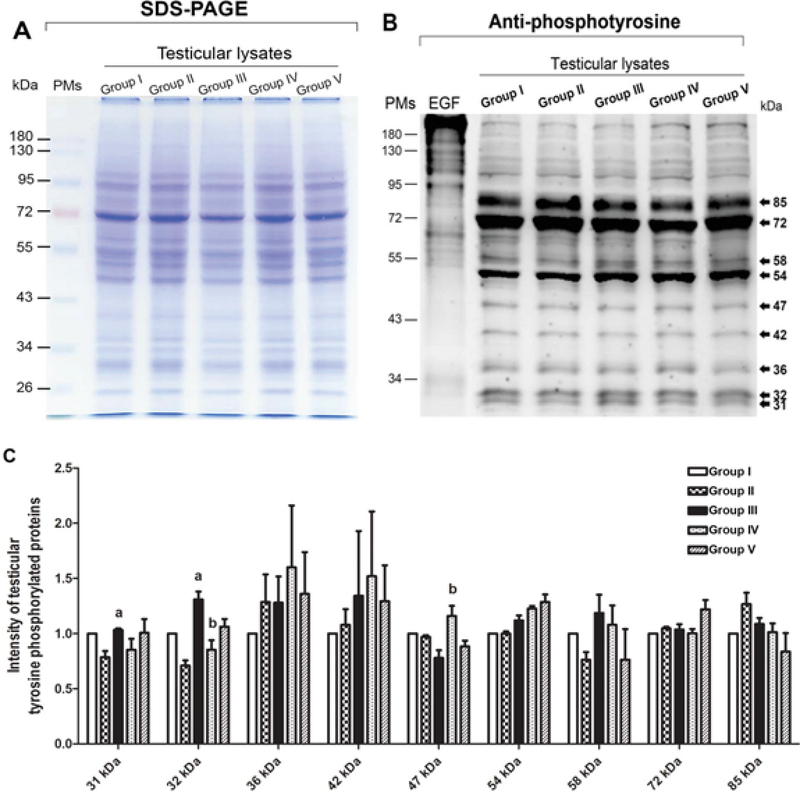
(A) SDS-PAGE showing the total testicular protein profiles. (B) Immuno-western blotting of tyrosine-phosphorylated proteins in testicular lysate. (C) The intensity of protein expression in the control and treated groups. PMs, pre-stained markers; EGF, epididymal growth factor (used as a positive control); a: p < 0.05 (compared with the control group); b: p < 0.05 (compared with the MTX group).

The graph presented in Figure 1A reveals that the total protein profiles in the testicular lysate were similar among all groups. In addition, the patterns of tyrosine-phosphorylated protein expression among the control and treated groups were all similar and included expressions at 31, 32, 36, 42, 47, 54, 58, 72, and 82 kDa (Figure 1B). However, the intensity of expression at 31 and 32 kDa in the MTX group was significantly higher than that in the control group (Figure 1C). Further, the expressions at 32 and 47 kDa in the preventive groups (IV and V) were significantly increased and decreased compared with those in the MTX group, respectively (Figure 1C).

### Effects of melatonin on testicular StAR protein expression in rats treated with MTX

**Figure 2:**
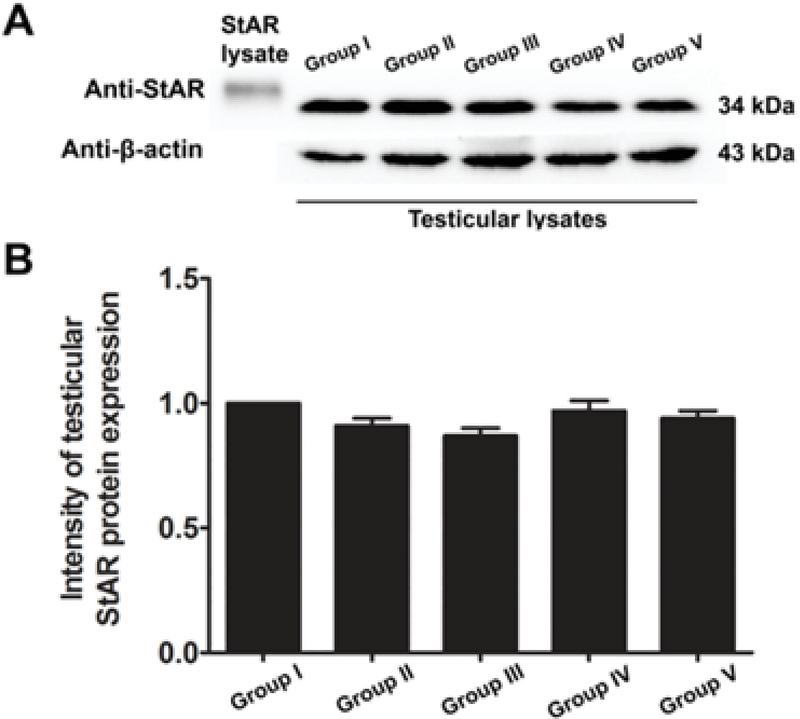
(A) Immuno-western blotting of StAR proteins in testicular lysate. (B) The ratio of StAR/β-actin in the control (I) and treated (II-V) groups. PMs, pre-stained markers; StAR lysate, positive control.

Western blot revealed that the StAR protein was expressed in the testicular lysate of all groups (Figure 2A). Using β-actin as an internal control, it was revealed that the intensity of testicular StAR protein expression was not significantly different among the groups (Figure 2B).

### Effects of melatonin and MTX on testicular caspase-3

**Figure 3:**
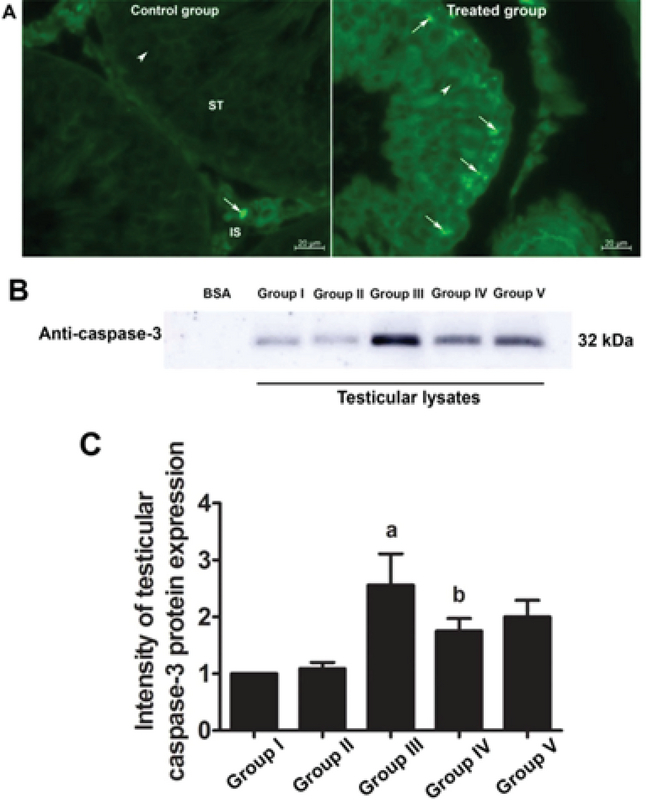
(A) Photographs showing the representative immunofluorescent staining of caspase-3 (FITC; green staining) in the seminiferous tubules of the control and treated groups. (B and C) Immuno-western blotting analysis of caspase-3 protein expression in testicular lysates of the control and treated groups. BSA, bovine serum albumin (used as the negative control); a: p < 0.05 (compared with the control group); b: p < 0.05 (compared with the MTX group); IS, interstitial space; ST, seminiferous tubule; arrows, caspase-3 positive; arrowheads, nucleus. 400X magnification.

The immunofluorescent staining results for caspase-3 revealed that the protein was localized in both the seminiferous epithelium and interstitial tissues of all groups (Figure 3A). This finding was also confirmed by western blotting (Figure 3B). However, the intensity of caspase-3 expression was much greater in all the treated groups than in the control group (Figure 3A). Figures 3B and 5C further revealed that the expression of caspase-3 in the MTX group was increased compared with that in the other groups. The intensity of caspase-3 expression was also lower in the preventive (IV and V) groups than in the MTX group (Figure 3C).

### Effects of melatonin on testicular MDA levels in rats treated with MTX

**Figure 4:**
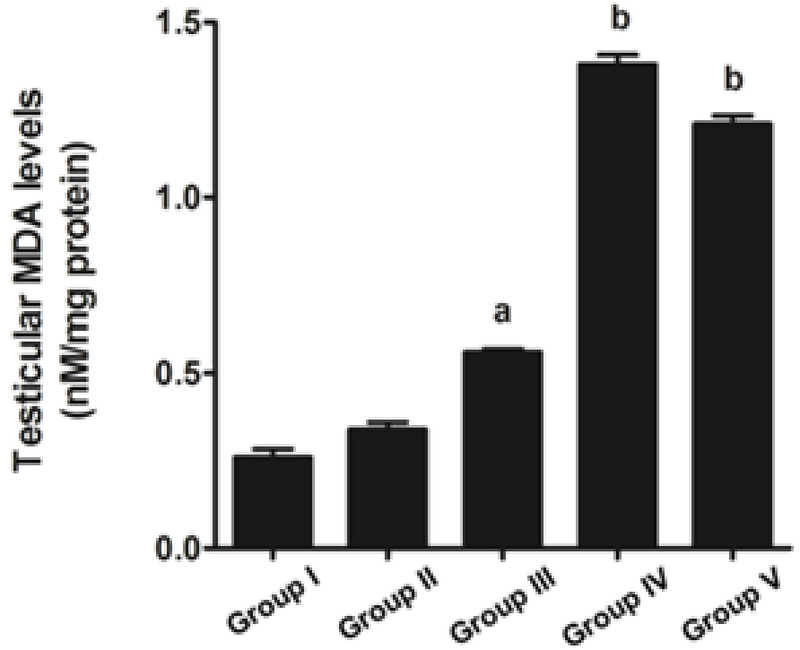
Graph showing the representative testicular MDA levels of the control and treated groups. Data are shown as Means ± SD. a: p < 0.05 (compared with the control group); b: p < 0.05 (compared with the MTX group).

Figure 4 shows a comparison of the testicular MDA levels among the control and treated groups. The results revealed that the MTX group underwent a significant increase in MDA level in the testicular lysate compared with the control and melatonin groups. Moreover, the MDA levels of the preventive and melatonin groups were significantly increased compared with that of the MTX group (Figure 4).

**Table 1:** The body weight and reproductive parameters in the study groups (n = 8/each)


**Parameters**		**Group I**	**Group II**	**Group III**	**Group IV**	**Group V**
**Body weight (g)**		409 ± 6	399 ± 6	408 ± 6	393 ± 7	372 ± 5
**Testes**						
	**Absolute weight (g)**	1.85 ± 0.03	1.88 ± 0.02	1.70 ± 0.04${}^{a}$	1.72 ± 0.06	1.81 ± 0.03
	**Relative weight (g 100 g ${}^{-1}$)**	0.45 ± 0.01	0.46 ± 0.01	0.43 ± 0.01	0.44 ± 0.01	0.49 ± 0.01${}^{b}$
**Epididymis plus vas deferens**						
	**Absolute weight (g)**	0.65 ± 0.02	0.61 ± 0.01	0.62 ± 0.01	0.60 ± 0.03	0.62 ± 0.02
	**Relative weight (g 100 g ${}^{-1}$)**	0.16 ± 0.004	0.15 ± 0.002	0.16 ± 0.004	0.15 ± 0.008	0.17 ± 0.003${}^{b}$
**Seminal vesicle and prostate gland**						
	**Absolute weight (g)**	2.04 ± 0.05	1.95 ± 0.06	1.87 ± 0.05	1.83 ± 0.07	1.68 ± 0.10
	**Relative weight (g 100 g ${}^{-1}$)**	0.50 ± 0.01	0.48 ± 0.02	0.47 ± 0.01	0.47 ± 0.01	0.45 ± 0.02
**Sperm concentration ( ×10${}^{6}$ cells mL ${}^{-1}$)**		77.44 ± 1.97	59.67 ± 2.17${}^{a}$	32.92 ± 2.14${}^{a,c}$	58.75 ± 1.28${}^{b}$	55.93 ± 2.57${}^{b}$
**Seminiferous tubular diameter (μm)**		253.96 ± 3.10	255.98 ± 3.89	221.89 ± 3.57${}^{a}$	224.55 ± 1.94	239.33 ± 2.42${}^{b}$
All data presented as Means ± SEM. a: p < 0.05 (compared with the control group), b: p < 0.05 (compared with the MTX group), c: p < 0.05 (compared with the melatonin group)						

## 4. Discussion

Chemotherapeutic agents have adverse effects on the male reproductive system, depending on the type and dosage of the drug used, and typically affect seminiferous epithelial cells (4). MTX is known as a low-risk gonadotoxic chemotherapeutic agent that affects the cell cycle via DNA and RNA synthesis inhibition, the inhibition of mitosis, and the deamination of proteins (26). In the present study, MTX was found to decrease the absolute testicular weight, the caudal epididymal sperm concentration, and the diameter of STs. These findings are supported by results from previous reports (14, 15, 27). In the present study, the significant decrease in testicular weight in the MTX group was correlated with the reduction of seminiferous epithelial height and diameter. This effect may have been caused by the increase in caspase-3 expression (Figure 3); our results thus indicate that MTX induces testicular apoptosis.

Our study further supports previous research that has shown the anti-inflammatory effect of melatonin via the nuclear factor erythroid 2-related factor 2 and nuclear factor kappa-light-chain-enhancer of activated B cell pathways in rat testes treated with MTX (28). Melatonin is further known to possess antioxidant capacities that can protect testicular injury in many animal models (29-31). In the present study, a significant decrease in sperm count was also associated with the histopathologies found in the testicular and caudal epididymis tissues. It should be obvious that MTX caused a reduction in seminiferous diameter and sperm mass within the epididymal lumen. These results might be due to the sensitivity of the mitotic rate of germinal epithelium to anticancer treatments (32). Although the level of testosterone in this study was not determined, the expression of the StAR protein revealed by western blotting showed no significant difference among the control and treated groups. Therefore, MTX may cause damage to the seminiferous epithelium via another mechanism, such as oxidative stress. In particular, the adverse effects of MTX may be caused by ROS, which create an imbalance between ROS production and antioxidant defense (33). The significantly increased testicular caspase-3 expression in the MTX group is consistent with an increase in mRNA expression reported by Sheikhbahaei and coworkers (34). In the present study, MTX significantly increased the testicular MDA level, which has also been reported previously (35, 36).

The antioxidant system in the testes consists of a number of antioxidant enzymes, including SOD, CAT, and GPX, as well as non-enzyme factors, such as vitamin C, vitamin E, and melatonin. Melatonin can regulate normal testicular function (17, 18). Moreover, it has anti-inflammatory, proliferative, and ROS scavenging effects (18, 19). Herein, we revealed that melatonin could reduce testicular damage in the melatonin group, a finding similar to that made by Wang and co-workers (36). In the present study, it was demonstrated for the first time that melatonin may protect testicular apoptosis by decreasing caspase-3 expression. However, the MDA level was increased significantly in the preventive groups, which is not in concordance with the results of some previous reports (37). We assumed that the melatonin dose used in this study for the stimulation of neuronal progenitor cell survival had no effect on MDA reduction (24). Tyrosine phosphorylation in the testes is essential for spermatogenesis, and pattern changes are associated with increased sperm concentration (4, 14, 38, 39). The increased expression of the tyrosine-phosphorylated protein at 31 and 32 kDa in the MTX-treated testes is similar to that reported by Iamsaard *et al.* (14). In addition, the expression of tyrosine-phosphorylated protein at 47 kDa was increased significantly in the melatonin groups compared with the MTX group. It is possible that this improvement in tyrosine-phosphorylated protein expression caused by melatonin treatment plays an important role in normal spermatogenesis.

## 5. Conclusion

Our results indicate that melatonin can increase seminiferous morphometry and epididymal sperm concentration. However, the testicular MDA levels in co-treated groups were not improved. Melatonin also reduces testicular caspase-3 expression and increases the expression of tyrosine-phosphorylated proteins in rats treated with MTX.

##  Conflict of Interest

The authors declare no conflicts of interests with regard to the present study.
